# Numerical and experimental analysis of a hybrid material acoustophoretic device for manipulation of microparticles

**DOI:** 10.1038/s41598-021-01459-0

**Published:** 2021-11-11

**Authors:** Alireza Barani, Peiman Mosaddegh, Shaghayegh Haghjooy Javanmard, Shahrokh Sepehrirahnama, Amir Sanati-Nezhad

**Affiliations:** 1 Department of Mechanical Engineering, Isfahan University of Technology, 84156-83111 Isfahan, Iran; 2 Applied Physiology Research Center, Cardiovascular Research Institute, Isfahan University of Medical Sciences, 81746-73461 Isfahan, Iran; 3 Mechanical Engineering Department, National University of Singapore, 9 Engineering Drive 1, Singapore, 117575 Singapore; 4 BioMEMS and Bioinspired Microfluidic Laboratory, Department of Mechanical and Manufacturing Engineering, University of Calgary, MEB 214 2500 University Drive NW, Calgary, AB T2N1 N4 Canada

**Keywords:** Biomedical engineering, Mechanical engineering, Lab-on-a-chip

## Abstract

Acoustophoretic microfluidic devices have been developed for accurate, label-free, contactless, and non-invasive manipulation of bioparticles in different biofluids. However, their widespread application is limited due to the need for the use of high quality microchannels made of materials with high specific acoustic impedances relative to the fluid (e.g., silicon or glass with small damping coefficient), manufactured by complex and expensive microfabrication processes. Soft polymers with a lower fabrication cost have been introduced to address the challenges of silicon- or glass-based acoustophoretic microfluidic systems. However, due to their small acoustic impedance, their efficacy for particle manipulation is shown to be limited. Here, we developed a new acoustophoretic microfluid system fabricated by a hybrid sound-hard (aluminum) and sound-soft (polydimethylsiloxane polymer) material. The performance of this hybrid device for manipulation of bead particles and cells was compared to the acoustophoretic devices made of acoustically hard materials. The results show that particles and cells in the hybrid material microchannel travel to a nodal plane with a much smaller energy density than conventional acoustic-hard devices but greater than polymeric microfluidic chips. Against conventional acoustic-hard chips, the nodal line in the hybrid microchannel could be easily tuned to be placed in an off-center position by changing the frequency, effective for particle separation from a host fluid in parallel flow stream models. It is also shown that the hybrid acoustophoretic device deals with smaller temperature rise which is safer for the actuation of bioparticles. This new device eliminates the limitations of each sound-soft and sound-hard materials in terms of cost, adjusting the position of nodal plane, temperature rise, fragility, production cost and disposability, making it desirable for developing the next generation of economically viable acoustophoretic products for ultrasound particle manipulation in bioengineering applications.

## Introduction

Microfluidic devices have enabled miniaturization of laboratory devices and allowed for a quick process of sample-to-answer with a minute amount of samples^1,2^. These devices have been used to separate^3–5^, sort^6–8^, focus^9,10^, pattern^11,12^, or wash bioparticles (e.g., cells and bacteria)^13^. These particles are driven by various stimuli including ultrasound waves^14–21^. The ultrasound manipulation of particles, commonly known as acoustophoresis^22–26^, is a contactless and label-free manipulation technique^27–29^, harmless to living cells compared to the other electrical or shear-based manipulation methods^30–33^. Acoustophoresis is a self-sufficient method without the need for additives to apply forces on particles. It typically operates at the micro- and nano-meter scale inside microchannels^34–38^.

Acoustophoretic microfluidic devices have been designed based on either Bulk Acoustic Wave (BAW) in a closed fluid cavity^39^ or Surface Acoustic Wave (SAW), generated by transducers through the bottom of the device^40^. Comparing the BAW and SAW, BAW-driven devices typically offer higher throughput and simpler designs of microfluidics^41–43^. In acoustophoretic devices, particles suspended in a fluid are subject to the so-called the acoustic radiation force, leading to scattering of the incident sound wave^44,45^. Under the acoustic radiation force, particles move toward either pressure nodes or anti-nodes of a standing wave depending on the size and material properties of the particles^46^. Running an ultrasound particle manipulation at the resonance frequency of the microchannel provides the highest localized acoustic radiation force^47,48^. The resonance frequency as a structural property is a function of material properties and shape of microchannels^49^. For microchannels made of materials with negligible structural damping (such as silicon), the vibration from the transducer propagates throughout the device with the least attenuation, while the vibration dies off quickly for materials with high damping factors (e.g. polydimethylsiloxane (PDMS) polymer)^50^. The optimal particle manipulation is obtained at the maximum energy density set up in the fluid cavity. At the fundamental frequencies of a piezoelectric transducer, the maximum input energy is generated and propagated through the device by elastic waves^51,52^.

Among various materials used for fabricating acoustophoretic devices, silicon and glass are the most widely used materials due to their appropriate acoustic properties, biocompatibility, and compatibility with high-precision fabrication techniques (e.g. lithography)^23,53–55^. However, these materials are relatively expensive and their manufacturing process is time-consuming and costly, meanwhile tuning the position of nodal line across the width of fluid cavity is still a challenge^56,57^. Leibacher et al.^58^ coated a PDMS layer within a silicon microchannel to interface the silicon wall and the fluid domain, wherein the position of the nodal line was relatively adjustable by controlling the thickness of PDMS. However, accurate PDMS coating was shown to be a very cumbersome process with a limited reproducibility. Low cost fully ploymeric microchannels can be fabricated by machining of Poly(methyl methacrylate) (PMMA) wherein the anti-symmetric acoustic pressure field in the fluid cavity can be made by attaching two piezoelectrics (or a sawed piezoelectric) below the PMMA body actuated under AC anti-phase signals. The theoretical and experimental studies have shown that in polymeric microchannels, the entire system (and not just the fluid cavity) should be optimally designed^59^. Sound-soft polymer microchannels deal with a large damping of waves with an adverse effect on the efficacy of particle manipulation, attenuating the wave propagation, decreasing the pressure amplitude, and increasing the rate of heat generation inside microchannels^50,60–65^. The temperature control in these systems requires a cooling mechanism using, for example, Peltier^59,66^ or aluminum heat sinks^61^. Despite these limitations, polymers still offer low manufacturing cost, high flexibility in design and assembly, easy disposal, and high biocompatibility, making them attractive for making acoustophoretic devices.

Considering the drawbacks of each of sound-hard and sound-soft materials for making acoustic microchannels^50,66–70^, this work presents a new fabrication method that eliminates the limitations of each sound-soft and sound-hard materials in terms of cost, adjusting the position of nodal plane, temperature rise, fragility, production cost and disposability. Aluminum and PDMS were selected as the materials used in this work for fabrication of a hybrid aluminum-PDMS microchannel. Aluminum and PDMS microchannels have been used in manufacturing of acoustofluidic microchannels^71,72^. In these microchannels, a plain PDMS layer was used to create a ceiling for the aluminum fluid cavity. Hence, PDMS is not along with the width of fluid cavity in the path of acoustic standing wave but the aluminum is in a direct contact with the fluid. Knowing that aluminum is a sound-hard material, it suffers from the drawbacks attributed to sound-hard materials. Aluminum has also limited biocompatibility which restricts its use in biophysical studies such as particle separation in blood. However, the choice of aluminum and PDMS is justified based on considerations of easy and scale-up production, reduced cost, and improved acoustophoretic functionality. To compare the behavior of three designs of aluminum, PDMS and hybrid aluminum-PDMS microchannels, computational modeling was implemented to simulate dynamic actuation of particles in these microchannels. Finally, the experimental results of particle manipulation in aluminum and hybrid aluminum-PDMS microchannels were compared to numerical data.

## Results and discussion

### Acoustophoretic actuation in an aluminum microchannel

**Figure 1:**
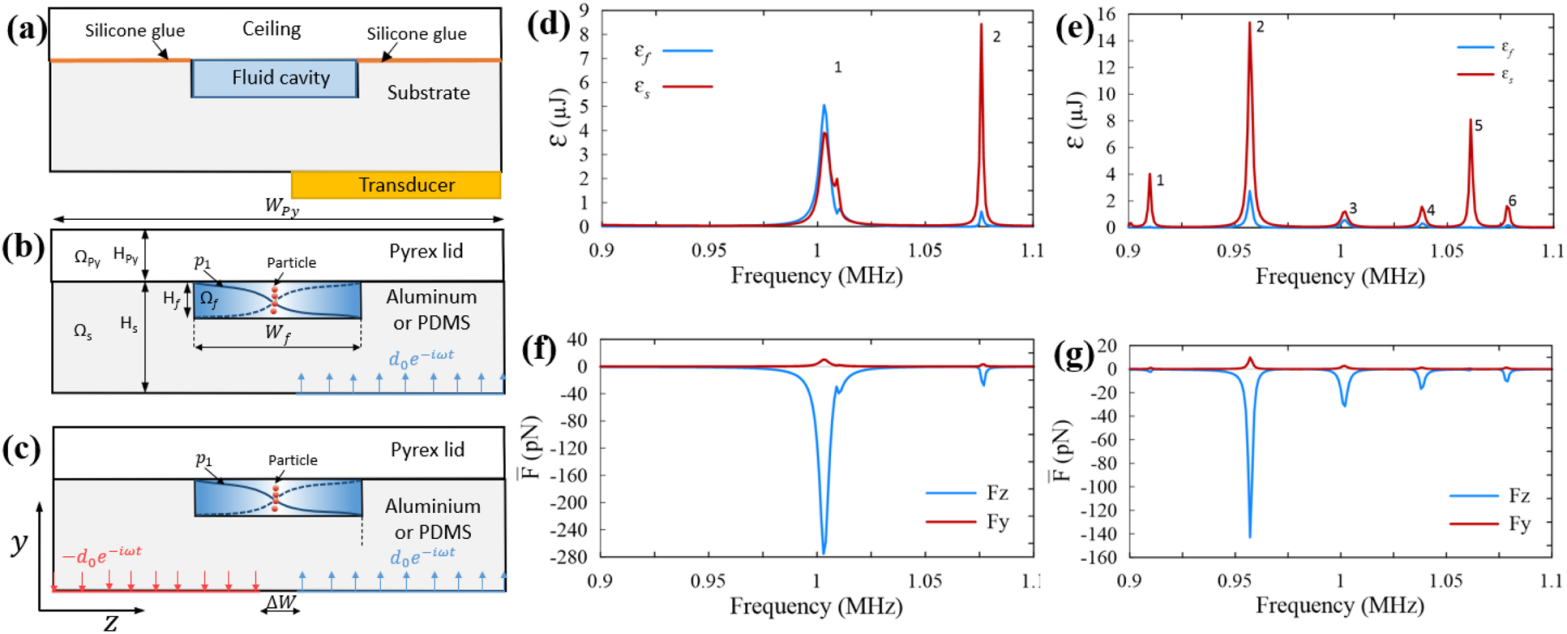
The acoustophoretic microfluidic system made of an aluminum microchannel used in this study. (**a**) Typical acoustophoretic device, (**b**) one-displacement actuation representing the one piezoelectric system, (**c**) two-displacement actuation representing the two piezoelectric system, (**d**) acoustic energy density of the fluid domain (blue curve) and the solid domain (red curve) in the two-displacement actuation. (**e**) is the same as (**d**) but for the one-displacement actuation, (**f**) average acoustic radiation force in the z direction (blue curve) and the y direction (red curve) in the two-displacement actuation. (**g**) is the same as (**f**) but for the one-displacement actuation.

One-displacement actuation represents a system with one piezoelectric stimulation (Fig. 1a,b), while two-displacement actuation represents the two piezoelectric system (Fig. 1c). Both displacement actuations were simulated here and the acoustic energy of their fluid domain (${\varepsilon }_{f}$) and solid domain (${\varepsilon }_{s}$) were calculated for frequency range of 0.9–1.1 MHz (as the resonance frequency of piezoelectric transducer). The frequency resonance of 1.07 MHz pertains to an infinitely hard-wall fluid cavity. The results of the acoustic energy density versus frequency show two main resonance frequencies for the two-displacement actuation (Fig. 1d) and six main resonance frequencies for the one-displacement actuation (Fig. 1e). These resonance frequencies are different from the resonance frequency of 1.07 MHz achieved for an infinitely hard-wall fluid cavity. The difference may be attributed to the damping effects. However, the resonance frequency of 1.076 MHz in the two-displacement actuation system is very close to the resonance frequency of 1.07 MHz. At frequency of 1.003 MHz in the two-displacement actuation system, most of the acoustic energy is concentrated in the fluid domain, although the solid domain has a larger area. This resonance might be a fluid-domain resonance^50^. All other resonances, even those very close to 1.07 MHz, have a higher acoustic energy in the solid domain than in the fluid domain. These resonances are so called the whole-system resonances^50^.

In the resonances of the fluid domain, the maximum energy is present in the fluid referring to the maximum damping occurring. The Q factor is calculated either by dividing the bandwidth of the fluid energy resonance curve at half of its maximum ($\text{Q}=f/\Delta f$) or estimated by weighted average damping coefficient. For domain i-th with stored energy ${\varepsilon }_{i}$ and damping coefficient ${\Gamma }_{i}$, the estimated Q factor of an acoustic device with n domains is calculated with Eq. (1), wherein the Q factor of the acoustic device in a fluid resonance is close to the Q factor of the fluid domain $(i.e., {Q}_{f}=1/2{\Gamma }_{f}=125)$.

1 $${Q}^{est}=\frac{1}{2}\frac{\sum_{i=1}^{n}{\varepsilon }_{i}}{\sum_{i=1}^{n}{\varepsilon }_{i}{\Gamma }_{i}}$$

In the aluminum microchannel, the frequency of 1.003 MHz in the two-displacement actuation system is a fluid domain resonance, knowing that its Q factor (Q = 193) is close to Q_f_ compared to other resonances. The Q factors of the acoustic device at resonances (so called whole-system resonances) other than the resonance of 1.003 MHz in the two-displacement actuation is much greater than Q factor of the fluid domain (Q = 125). Figure 1d,f confirm that most of the acoustic energy is accumulated in the solid domain at the higher resonances.

The spatial average of the acoustic radiation force components applied on 25 µm particles are plotted in Fig. 1f,g. In all the resonances, the spatial average acoustic radiation force in the z direction (${\overline{F} }_{z}$) conducts particles toward the nodal plane, with a value much larger than the force component in the y direction (${\overline{F} }_{y}$). The greater the ${\overline{F} }_{z}$ compared to ${\overline{F} }_{y}$, the better the acoustophoretic property of the device, quantified by the ratio of ${\overline{F} }_{z}$ to ${\overline{F} }_{y}$ defined as $R=-\frac{{\overline{F} }_{z}}{{\overline{F} }_{y}}$^50^. The resonances with higher R values offer stronger horizontal acoustic radiation forces. R values for each of the resonances in both one-displacement and two-displacement actuation systems are presented in Table 1. The R value of the resonance #1 in the two-displacement actuation system has the highest value among R values at other resonance frequencies, wherein the acoustic energy is focused on the fluid domain, generating a higher acoustic pressure amplitude and stronger acoustic radiation force in the z direction.

**Table 1:** The value of resonance frequency f, Q factor, acoustic energy density of the fluid $({E}_{f})$, average acoustic radiation force in the z direction (${\overline{F} }_{z}$), and average acoustic radiation force in the y direction (${\overline{F} }_{y})$ shown in Fig. 1 for the aluminum microchannel modeled in this work.

Resonance number (#)	f (MHz)	$\text{Q}$	E_f_ (Pa)	${\overline{\text{F}}}_{{\text{z}}}$ (pN)	${\overline{\text{F}}}_{{\text{y}}}$ (pN)	R
**Two-displacement actuation model**						
1	1.003	193	24	276	10.3	26.8
2	1.076	703	3	36.9	4.78	7.7
**One-displacement actuation model**						
1	0.91	414	0.3	2.4	1.1	2.18
2	0.957	399	13	142.95	9.89	14.5
3	1.002	267	2.5	31.56	2.65	11.9
4	1.038	358	1.5	16.79	1.23	13.7
5	1.061	530	0.2	1.16	0.42	2.8
6	1.079	450	0.9	10.43	1.1	9.5

**Figure 2:**
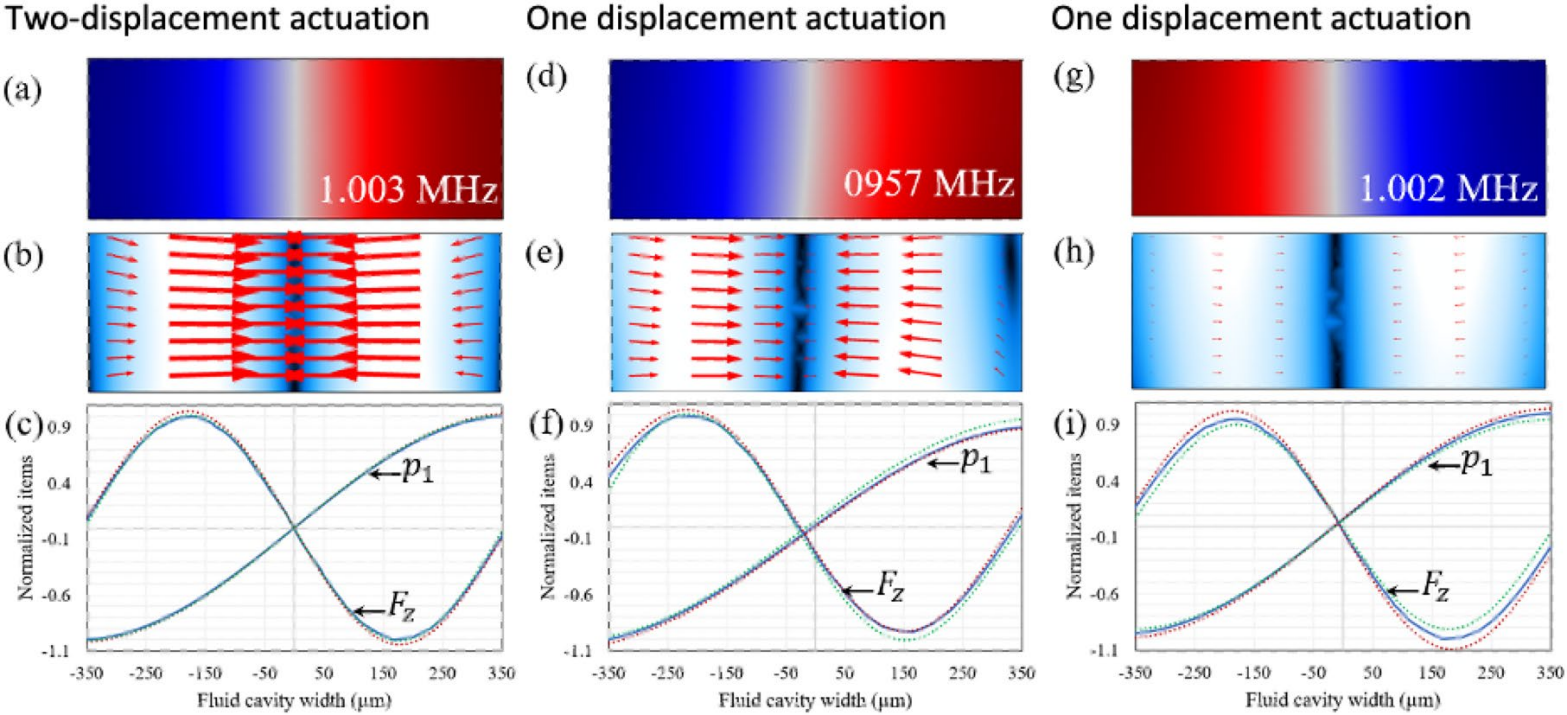
The acoustic pressure fields and resonances of aluminum microchannels. (**a**) The surface plot of the anti-symmetric acoustic pressure (red (− 45 kPa) to blue (45 kPa)), (**b**) the surface and vector plots of the acoustic radiation force (black (0 pN) to white (500 pN)), and (**c**) the line plot of the horizontal acoustic radiation force and the corresponding acoustic pressure along three lines passing the fluid cavity’s cross section in three different heights (red dotted curve for the top line (0.95 H_f_), blue solid curve for the center line (0.5 H_f_) and green dotted curve for the bottom line (0.05 H_f_)) for the resonance frequency of 1.003 MHz for two-displacement actuation. (**d**) the surface plot of the acoustic pressure (blue (− 17 kPa) to red (15 kPa)), (**e**) the surface and vector plots of the acoustic radiation force (black (0 pN) to white (250 pN)), and (**f**) the line plot of the acoustic pressure and horizontal acoustic radiation force for the resonance frequency of 0.957 MHz for one-displacement actuation. (**g**) the surface plot of the acoustic pressure (red (− 41 kPa) to blue (42 kPa)), (**h**) the acoustic radiation force (black (0 pN) to white (52 pN)), and (**i**) the line plot of acoustic pressure and horizontal acoustic radiation force for the resonance frequency of 1.002 MHz for one-displacement actuation.

The simulation results show that anti-symmetric pressure fields are present at all the resonances of the two-displacement actuation model, but the wave node where pressure is zero is not exactly located in centerline of the fluid cavity in the one-displacement actuation model. Figure 2 shows anti-symmetric pressure fields for two-displacement actuation’s resonance of 1.003 MHz (Fig. 2a) and one-displacement actuation’s resonances of 0.957 (Fig. 2d) and 1.002 MHz (Fig. 2g), respectively. The nodal line position is not exactly in centerline of the fluid cavity in resonances of the one-displacement actuation. This deviation from infinitely hard wall fluid cavity is because of the effect of wall material on wave reflection in the fluid cavity. However, the off-center node observation is not seen for resonances of the two-displacement actuation because of its symmetrically positioned displacement actuation. On the other hand, it is experimentally desired to actuate an acoustic device by one piezoelectric actuator as the two piezoelectric actuators in two-displacement actuation system needs to be located truly symmetric relative to the centerline of the fluid cavity and accurately driven by a function generator.

The distance of the off-center nodal line to the axial centerline of the microchannel in the one-displacement is primarily dependent on the resonance frequency. For instance, the nodal line in the resonance of 1.002 MHz (Fig. 2g) is very close to the centerline compared to the resonance of 0.957 MHz (Fig. 2d). The pressure field of these resonances can be used for acoustophoretic collection of microparticles with positive acoustic contrast factor on the centerline of the microchannel, while ignoring a small deviation of the nodal line from the centerline. The direction of the acoustic radiation force on a 25 µm polystyrene sphere for the three resonances of 1.003 (Fig. 2b), 0.957 (Fig. 2e) and 1.002 MHz (Fig. 2h) show that the net acoustic force is very prone toward the centerline. However, the arrows representing the acoustic radiation force are not perfectly horizontal as the acoustic radiation force still has a component in y direction $({F}_{y})$ due to the difference in the materials of ceiling and floor (substrate) of the fluid cavity. This leads to a different leakage of the acoustic energy into the top and bottom surface of the fluid cavity. For acoustophoretic systems with high R values, the net acoustic forces are more prone to the horizontal direction.

The acoustic pressure (half-wavelength sinusoidal curves) and the horizontal component of the acoustic radiation force on a 25 µm polystyrene sphere (full-wavelength sinusoidal curve) along the width of the fluid cavity in three different heights (i.e., 0.05 H_f_, 0.5 H_f_ and 0.95 of H_f_, respectively represented in green dotted, blue solid and red dotted curve) are shown in Fig. 2c for the two-displacement actuation system (resonance frequency of 1.003 MHz) and in Fig. 2f,i for the one-displacement actuation system (respectively for resonance frequencies of 0.957 and 1.002 MHz in the aluminum microchannel). The results show that the difference between the values of both acoustic pressure and acoustic force at different heights of the microchannel is less in the two-displacement actuation system compared to the one-displacement actuation system. This is in agreement with the ideal infinitely hard wall fluid cavity, wherein there is no difference between the acoustic pressure and the acoustic radiation force in different heights of the fluid cavity. The difference between the acoustic pressure and the acoustic force in the one-displacement actuation is attributed to its anti-symmetric actuation in which the generation of anti-symmetric pressure field relies on multiple wave reflections of the cavity walls of different materials. Nevertheless, in both one-displacement and two-displacement actuation systems, the acoustic pressure and the acoustic force are still identical to that of the infinitely hard wall fluid cavity, thanks to the use of an aluminum microchannel with hard-like acoustic material properties.

### Acoustophoretic actuation in PDMS microchannels

**Figure 3:**
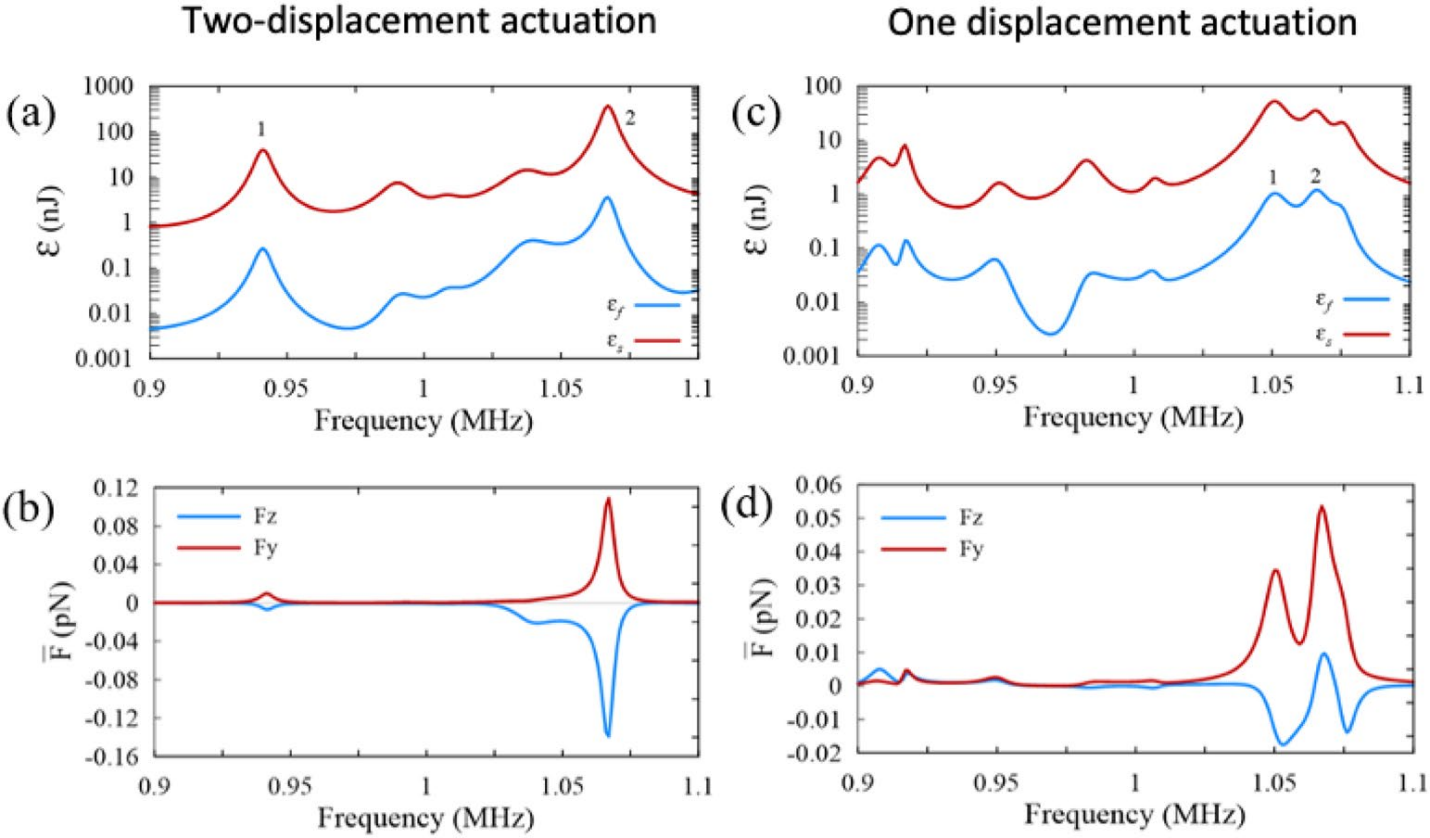
The plot of acoustic energy density and average acoustic radiation force of polydimethylsiloxane (PDMS) microchannels. (**a**) The semi-log plot of the acoustic energy density for the fluid domain (blue curve) and the solid domain (red curve), and (**b**) the average acoustic radiation force in the z direction (blue curve) and in the y direction (red curve) under the two-displacement actuation. (**c**) and (**d**) are the same as (**a**) and (**b**) but under the one-displacement actuation.

Similar to the aluminum microchannel, the PDMS microchannel with a ceiling of Pyrex lid is actuated by both two-displacement and one-displacement actuations. Considering the permanent bonding of the Pyrex lid and PDMS microchannels to the PDMS layer via an oxygen plasma activation in the experimental tests, the silicone glue layer does not exist and is not considered in the simulations. The dimensions of the PDMS microchannel are given in Table 2, designed based on the material properties of PDMS^50^ and with the aim of working with the resonance frequency of 1.07 MHz. The specific acoustic impedance of PDMS is $1.04\times {10}^{6}$ kg m^−2^ s^−1^, which is close to the specific acoustic impedance of the water ($1.49\times {10}^{6}$ kg m^−2^ s^−1^), meaning that the acoustic energy reflected from the fluid cavity’s walls is weak compared to the aluminum microchannel, generating smaller pressure amplitude and lower acoustic radiation forces. The acoustic energy density and average acoustic radiation force on a 25 µm polystyrene particle of two-displacement actuation of PDMS microchannel are shown in Fig. 3a,b, while the corresponding plots of one-displacement actuation are shown in Fig. 3c,d. The acoustic energy in the solid domain of PDMS microchannel is much higher than the one in the fluid domain. The two-displacement actuation is preferred to create anti-symmetric pressure field in the PDMS microchannel. The corresponding average acoustic radiation force versus frequency is shown in Fig. 3b,d. The force components are slightly different from each other, given the small R value and weak acoustophoretic properties. For one-displacement actuation model, it is noticeable that the wave reflection from the cavity walls plays an important role in generating standing waves in the absence of one of the displacement actuations. Therefore, standing acoustic field in PDMS microchannels is produced by either surface acoustic waves^73^ or lamb waves^74^. Acoustic standing waves are made by superposition of incident waves stemmed from either sides through the bottom surface while the walls have minimum contribution to reflecting the waves.

**Table 2:** Geometrical parameters of different microchannels used in this acoustophoretic study.

Parameter	Symbol	Value (mm)
Height	H_Py_	1
Height	H_s_	2
Actuator gap	ΔW	0.1
Width	W_*f*_	0.7
Height	H_*f*_	0.3
**Aluminum microchannel**		
Width	W_Py_	9.7
**PDMS microchannel**		
Width	W_Py_	3.06
**Hybrid aluminum-PDMS microchannel**		
Width	W_Py_	23.58
Width	W_PDMS_	2.58

**Figure 4:**
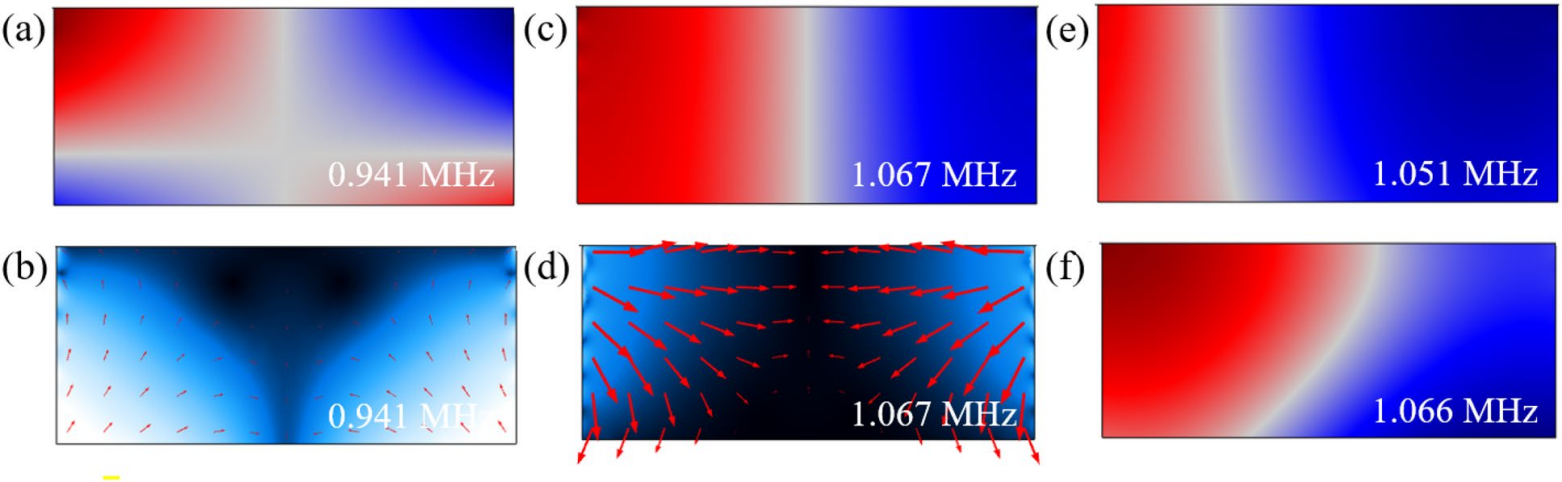
Numerical simulation of two-displacement and one-displacement actuations in the PDMS microchannel. (**a**) Surface plot of acoustic pressure (red (− 350 Pa) to blue (350 Pa)) and (**b**) surface and vector plot of the acoustic radiation force (from black (0 pN) to white (0.001 pN)) for the resonance frequency of 0.941 MHz under the two-displacement actuation. (**c**) Surface plot of acoustic pressure (blue (− 3.2 kPa) to red (3.2 Pa)) and (**d**) surface and vector plot of the acoustic radiation force (black (0 pN) to white (3.5 pN)) for the resonance frequency of 1.067 MHz under the two-displacement actuation. (**e**) and (**f**) surface plot of acoustic pressure, respectively, for the resonance frequency of 1.051 MHz (blue (− 2.9 kPa) to red (2.9 kPa)) and 1.066 MHz (blue (− 1.5 kPa) to red (5.2 kPa)) under the one-displacement actuation.

Q factors and R values of different resonances of the PDMS microchannel are shown in Table 3. The highest Q factor and R values are related to the resonance #2 of the two-displacement actuation, although these values are much smaller than their counterparts in the aluminum microchannel. The R values in other resonances are less than 1 which implies that ${\overline{F} }_{y}$ is greater than ${\overline{F} }_{z}$, causing weak acoustophoretic actuation in z the direction (Fig. 4a–d). Knowing that ${\overline{F} }_{y}$ pushes the particles toward either top (Fig. 4b) or bottom of the fluid cavity (Fig. 4d), this property can be used for applications like particle sedimentation.

**Table 3:** The values of resonance frequency (f), Q factor, acoustic energy density of the fluid ($${E}_{f})$$, average acoustic radiation force in the z direction ($${\overline{F} }_{z})$$, average acoustic radiation force in the y direction ($${\overline{F} }_{y})$$ and R shown in Fig. 3 for the PDMS microchannel.

Resonance	*f* (MHz)	$Q$	*E*_*f*_ (Pa)	$\overline{F}_{z}$ (pN)	$\overline{F}_{y}$ (pN)	R
**Two-displacement actuation model**						
1	0.941	149	0.001	0.007	0.01	0.71
2	1.067	187	0.017	0.139	0.109	1.28
**One-displacement actuation model**						
1	1.051	83	0.005	0.016	0.034	0.45
2	1.066	65	0.006	0.007	0.051	− 0.13

Characterizing the resonances of the PDMS microchannel show that the resonance of 0.941 MHz has an acoustic pressure field with two nodal lines crossing each other. This field consists of two anti-symmetric acoustic pressure fields in y and z the directions while the dominant direction of the acoustic radiation force is toward the center and top of the microchannel. The resonance frequency of 1.067 MHz with the highest R value showed an anti-symmetric acoustic pressure field by which an acoustic radiation force is generated toward the center and bottom of the microchannel. ${\overline{F} }_{y}$ in larger scale relative to ${\overline{F} }_{z}$ redirects the total acoustic radiation force toward the top and bottom of the microchannel. The force component in the y direction is generated due to the difference in acoustic properties of the Pyrex lid and PDMS.

While the resonance frequencies at the two-displacement actuation contain anti-symmetric acoustic pressure fields with zero acoustic pressure in center, the resonances at the one-displacement actuation lack this pressure field (Fig. 4e,f). The off-center nodal line in one-displacement actuation resonances can be attributed to the high transmission coefficient at the PDMS/water interface and the high damping coefficient of PDMS, the acoustic wave energy partially enters the cavity wall and dissipates while a small portion of this energy reflects. The reflected waves interfere with the incident waves and generate a standing wave with the off-center nodal line. As seen in Fig. 4f, this behavior also occurred for the resonance #2 of the one-displacement actuation system, although its nodal line is not vertical and has a negative R value. Smaller R values in the one-displacement actuation confirms that this type of actuation offers weak acoustophoretic property in microchannels made of sound-soft materials.

### Acoustophoretic actuation in a bi-material PDMS-aluminum microchannel

**Figure 5:**
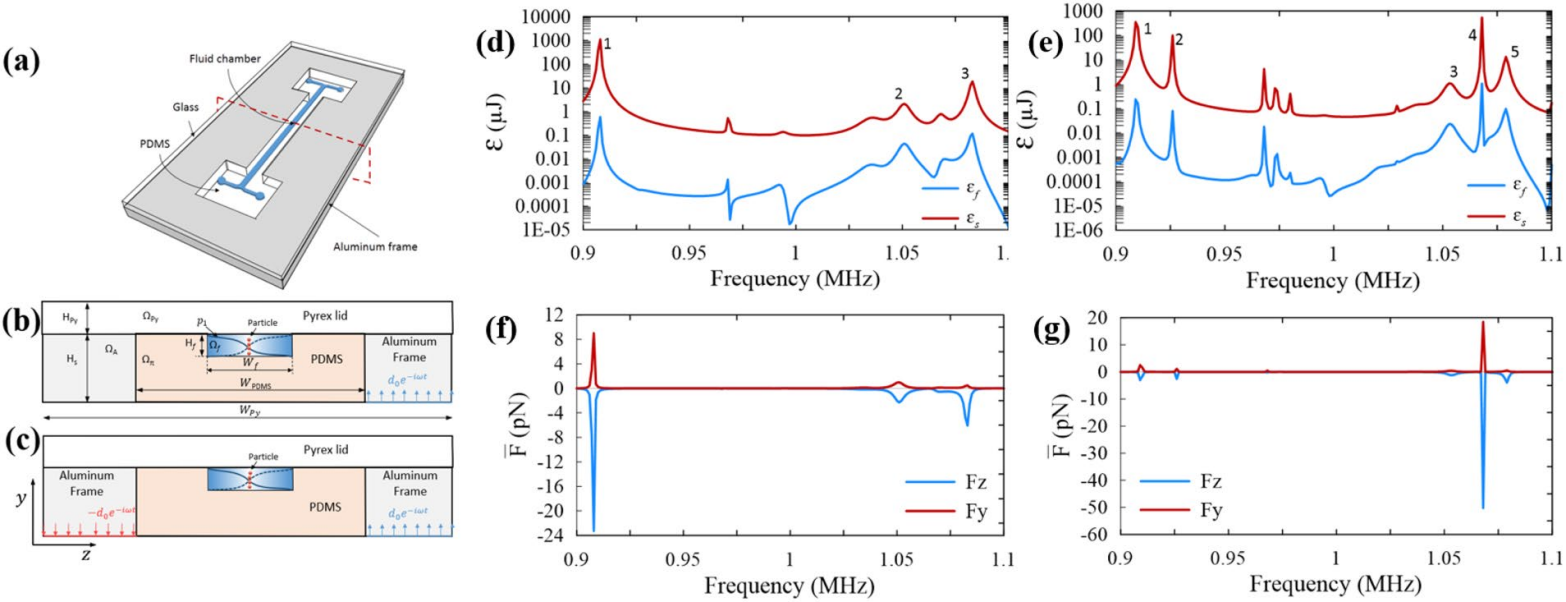
The design and acoustophoretic simulation of the hybrid aluminum-PDMS microchannel. (**a**) Hybrid aluminum-PDMS microchannel, (**b**) one-displacement actuation representing the one piezoelectric system, (**c**) two-displacement actuation representing the two piezoelectric system, (**d**) semi-log plot of acoustic energy density of the fluid domain (blue curve) and the solid domain (red curve) of the two-displacement actuation, (**e**) is the same as (**d**) but for one-displacement actuation, (**f**) average acoustic radiation force in the z direction (blue curve) and in the y direction (red curve) under the two-displacement actuation, (**g**) is the same as (**f**) but for one-displacement actuation.

The numerical data show that PDMS microchannels (PDMS channel walls) only offer a reliable acoustophoretic property when it is actuated by the two-displacement actuation model, although they still have a small acoustic energy density and small R values compared to aluminum microchannels. To address this challenge, a hybrid aluminum-PDMS microchannel is examined to get benefit from the acoustic properties associated with both aluminum and PDMS. This hybrid microchannel is made of aluminum and PDMS with the ceiling of Pyrex lid (Fig. 5a). The bonding between the Pyrex lid and the PDMS layer is performed by an oxygen plasma activation while there is no contact between the aluminum and the Pyrex lid. The aluminum domain is a frame around the PDMS domain with a polymer-to-metal connection. The fluid cavity is formed in PDMS while the displacement actuations are on aluminum frame boundaries (Fig. 5b,c). Based on the dimensions of the microchannel (Table 2), the acoustophoretic behaviors within the microchannel under one-displacement (Fig. 5b) and two-displacement (Fig. 5c) actuations were simulated. The results show three most-important resonance frequencies for the two-displacement actuation (Fig. 5d) and five most-important resonance frequencies for the one-displacement actuation (Fig. 5e).

The acoustic energy in the solid domain for all resonances is larger than in the fluid domain. Hence, these resonances can be defined as the whole-system resonances. The Q factor and R values of all resonances for the hybrid microfluidic system are shown in Table 4. Q factors of the hybrid microchannel are higher than the PDMS microchannel, indicating a system with less energy dissipation. Also, the acoustic energy densities in the hybrid microchannel are relatively smaller than in the aluminum microchannel. Therefore, the hybrid microchannels made of sound-soft and sound-hard materials have a superior acoustophoretic performance compared to the microchannels fully made of polymers. It is also confirmed that higher acoustic radiation forces and higher R values are obtained in the hybrid aluminum-PDMS microchannel (Fig. 5f,g) compared to PDMS microchannels (Fig. 3b,d).

**Table 4:** The values of f, Q factor, ${E}_{f}$, ${\overline{F} }_{z}$, (${\overline{F} }_{y})$, and R parameters shown in Fig. 5d,f for the hybrid aluminum-PDMS microchannel.

Resonance	f (MHz)	$\text{Q}$	E_f_ (Pa)	${\overline{\text{F}}}_{{\text{z}}}$ (pN)	${\overline{\text{F}}}_{{\text{y}}}$ (pN)	R
**Two-displacement actuation model**						
1	0.908	698	2.75	23.3	9	2.6
2	1.051	178	0.2	2.28	1	2.28
3	1.083	423	0.54	6.1	0.48	12.6
**One-displacement actuation model**						
1	0.909	505	1.12	3	2.5	1.2
2	0.926	926	0.37	2.6	1.1	2.2
3	1.053	181	0.11	1.2	0.4	2.8
4	1.068	1068	4.9	50.2	18.5	2.7
5	1.079	490	0.45	3.9	0.5	7.6

**Figure 6:**
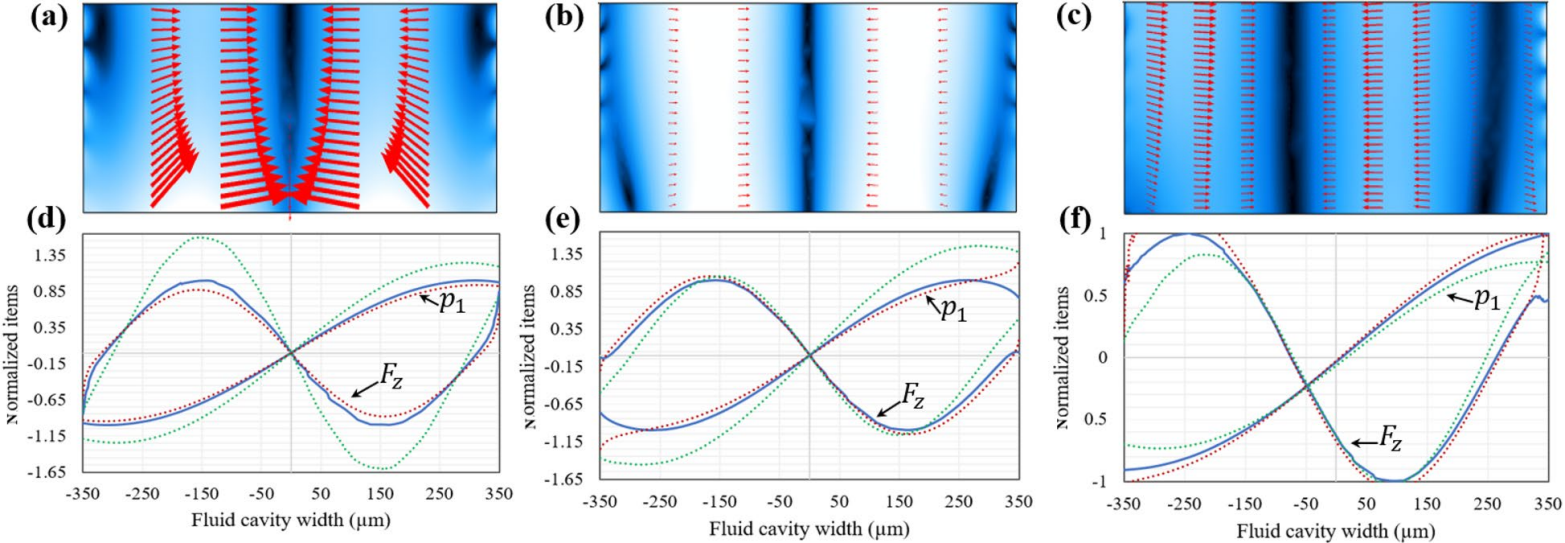
Numerical acoustophoretic simulation of the hybrid aluminum-PDMS microchannel. (**a**) Surface and vector plot of the acoustic radiation force (black (0 pN) to white (72 pN)) for the resonance frequency of 0.908 MHz. (**b**) and (**c**) are the same as (**a**) but respectively plotted for the resonance frequency of 1.083 MHz (surface plot represent black (0 pN) to white (11 pN)) and the resonance frequency of 1.079 MHz (surface plot represent black (0 pN) to white (22.5 pN)). (**d**) line plot of the horizontal acoustic radiation force and corresponding acoustic pressure for the resonance frequency of 0.908 MHz, (**e**) and (**f**) are the same as (**d**) but respectively plotted for the resonance frequency of 1.083 MHz and the resonance frequency of 1.079 MHz.

The results of acoustophoretic simulation obtained at the cross section of the fluid cavity (Fig. 6a–c) show that the acoustic radiation force in the z direction on a 25 µm polystyrene particle is higher than its component in the y direction and toward the center of fluid cavity under both one-displacement and two-displacement actuations. The results of the two-displacement actuation (Fig. 6a,b) show that the nodal line is positioned in the center of fluid cavity. The overall acoustic radiation force at the resonance frequency of 0.908 MHz is higher than the values in the other two resonance frequencies. ${p}_{1}$ and ${F}_{z}$ curves (respectively in the half-wavelength and full-wavelength sinusoidal shapes) in three different heights of fluid cavity (i.e., 0.05 H_f_, 0.5 H_f_ and 0.95 of H_f_, respectively represented in green dotted, blue solid and red dotted curve) are plotted at two different resonance frequencies of 0.908 MHz (Fig. 6d) and 1.083 MHz (Fig. 6e). The acoustophoretic properties of the hybrid microchannel at both resonances are superior to their counterparts for the PDMS microchannel with the two-displacement actuation system. The hybrid microchannel manipulates particles to vertical nodal lines with higher R values than the PDMS microchannel.

On the other hand, for the one-displacement actuation system, the largest R value in the hybrid microchannel is related to the resonance frequency of 1.079 MHz (Fig. 6c). The acoustic radiation force in this system is toward an approximately vertical nodal line. This resonance frequency of the hybrid microchannel offers a better acoustophoretic property than the PDMS microchannel and the other resonance frequencies of the two-displacement actuation. Therefore, adding a sound-hard material to polymeric microchannels offer better pressure fields for acoustophoretic applications, while there is no appropriate resonance under the one-displacement actuation in the PDMS microchannel. In the one-displacement actuation system, the nodal plane is not positioned in the centerline, but the shape of pressure and the acoustic radiation force curves are sinusoidal with a slight difference in different heights of the fluid cavity (Fig. 6f). Both one-displacement and two-displacement actuations in the hybrid aluminum-PDMS microchannel have shown to work with appropriate resonances, wherein strong acoustophoretic function can be achieved even with one piezoelectric actuator located under the microchannel.

### Experimental acoustophoretic manipulation of bead particles and cells

**Figure 7:**
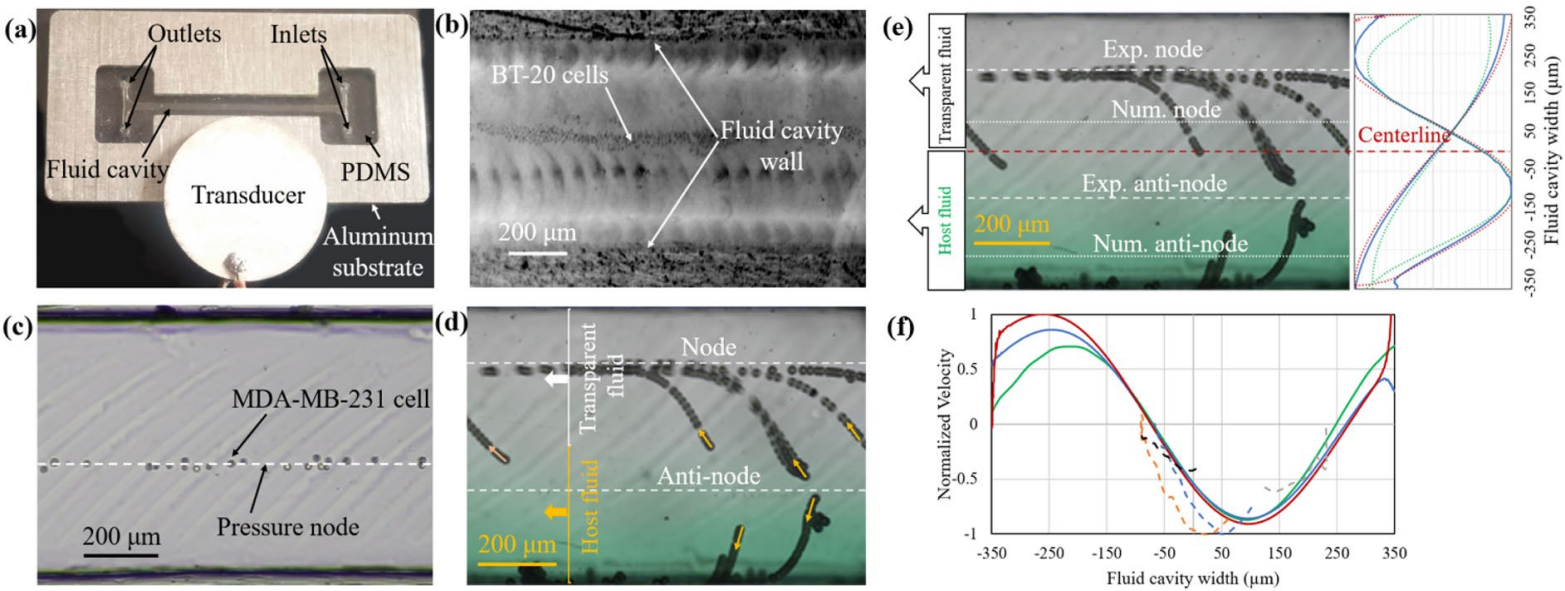
Experimental set-up and the testing results for acoustophoretic actuation of breast cancer cells and bead particles. (**a**) The fabricated hybrid aluminum-PDMS microchannel, (**b**) acoustic actuation of BT-20 cancer cells (average diameter: 17.5 µm) toward the nodal line in the aluminum microchannel, (**c**) acoustic actuation of MDA-MB-231 cancer cells toward the nodal line in the hybrid aluminum-PDMS, (**d**) acoustic actuation of polystyrene particles and their trajectory in direction of thin yellow arrows. (**e**) the numerical and experimental node and anti-node positions relative to the centerline. (**f**) normalized velocity along the width of the fluid cavity.

To experimentally evaluate predictivity of the acoustophoretic numerical model developed above, acoustic particle actuation was conducted on fabricated acoustophoretic devices (Fig. 7a) with one piezoelectric transducer located on one side of fluid cavity (i.e., one-displacement actuation). First, we assessed whether the aluminum device can actuate BT-20 cancer cells toward the nodal plane designed to be at the middle of the microchannel. After injection of the cells suspended in phosphate buffer saline (PBS), the setup was left at rest to eliminate any liquid flow. The excitation frequency was then set to 1.04 MHz close to the resonance frequency obtained from the numerical model. At this resonance frequency, a voltage of 26.6 Vp was applied to create the standing wave. The results show that the cells migrated quickly toward the middle of the microchannel (Fig. 7b), in agreement with the numerical data. Although the quality of images was adversely affected by opacity of the aluminum and surface roughness of the fabricated cavity, the cells are visible for accurate particle tracing.

The performance of the hybrid aluminum-PDMS microchannel was then assessed by the actuation of MDA-MB-231 breast cancer cells suspended in PBS. The acoustic setup was run in the frequency range of 0.8–1.2 MHz. At the frequency of 0.85 MHz, cells travel toward the nodal line. This frequency is around the resonance frequency #1 (0.909 MHz) of the one-displacement actuation obtained from the numerical model. The difference may be attributed to the errors stemmed from manufacturing and assembly errors as well as uncertainties in material properties. The cells successfully travelled to the desired position of the pressure node to form a pearl-chain (Fig. 7c). The nodal lines in Fig. 7b,c are not positioned at the center of the fluid cavity, in agreement with the numerical data. Also, because of the damping effects and one side actuation of particles, there is a phase lag between the incident wave and the reflected one, putting a nodal line to an off-center position.

It is noted that in the hybrid aluminum-PDMS microchannel, the trace of milling cutter was left on the aluminum mold and therefore replicated on the PDMS surfaces (Fig. 7c,d). Although the use of silicon mold made by lithography reduces dimensional errors and suppress the difference between theoretical and empirical results, the cost of mold fabrication drastically increases compared to the aluminum mold. The hybrid microchannel has the potential to be produced in mass scale, therefore the fabrication cost of the aluminum mold in mass scale production is negligible.

Off-center nodal lines are appealing for acoustic-based particle separation in flow-based liquid manipulation systems. Upon introducing two aqueous liquids (one containing 25 µm polystyrene particles suspended in host fluid containing 2% volume fraction of green color and another one with no particle suspended in transparent fluid) flowed in parallel with flow rate of 10 µL min^−1^ into the hybrid aluminum-PDMS microchannel and at the condition of off-center nodal line (located in the transparent fluid), the acoustic radiation force can quickly propel particles from the host fluid to the transparent fluid. This technique can be performed in resonances with off-center nodal lines (Fig. 7d,e). Those particles above the anti-node travel to the nodal line in the transparent fluid and those below the anti-node travel to the nodal line under the anti-node located near the wall. This type of particle separation is effective and feasible within the hybrid aluminum-PDMS microchannel while it is difficult to implement within microchannels made of sound-hard materials, knowing that their off-center nodal lines are very close to the centerline of the fluid cavity. The resonance frequency of 1.079 MHz has a pressure field with an off-center nodal line.

Because of the manufacturing errors, there was a slight difference between the numerical and experimental data in determining the location of nodal line, wherein the distance between the nodal line and the center line was larger in the experiment than the numerical model. This technique is helpful for separating particles from their host fluid. A green food dye was added to the host fluid to make a contrast between the host green fluid and the transparent fluid, although the interface between these two fluids is not very steep due to the gradual diffusion of green color into the transparent fluid.

The difference between theoretical and experimental results in particle separation is quantified in Fig. 7e for the hybrid aluminum-PDMS microchannel under the one-displacement actuation system. The node of pressure field at 1.079 MHz resonance frequency in the numerical model is located about 70 µm away from the centerline. This distance is 180 µm in experimental testing, which is 110 µm more than the results obtained from the numerical model. The difference between the theoretical and experimental data is attributed to the manufacturing and assembly errors. Although these errors can be suppressed by employing lithography techniques for manufacturing of the mold and the frame, one of the main purposes of this study is to develop low cost but high performance acoustofluidic microchannels manufactured with conventional and low-cost methods. It is shown that despite the manufacturing errors, the hybrid microchannels has a good performance in collecting polystyrene particles at the nodes. Figure 7e shows that the acoustic radiation force is zero in the node and anti-node but particles above the anti-node move toward the node located in the transparent fluid while those initially positioned below the anti-node travel to a node located beyond the limit of the microchannel at the lower wall. The separation efficiency is determined to be about 76% but it could be improved if a parallel transparent fluid is added next to the lower wall or a microchannel working with a λ/4 mode is employed. Both techniques can be easily implemented by manufacturing of hybrid microchannels.

One advantage of the hybrid microchannel over those fully acoustic-hard material microchannel is the control of node position. Node position can be adjusted by either frequency modulation or controlling the thickness of the PDMS wall. As demonstrated in Supplementary Video 1, altering the frequency replaces the nodal line position. This is a drastic result which cannot be easily achieved in microchannels made of acoustic-hard materials. As seen in Video 1, the node position moves to a different position along the width of the fluid cavity upon changing the frequency, helping the user to adjust the node position to the desired place in respect to the wall and to control particle manipulation toward a desired destination. Although altering the frequency implies moving away from the resonance frequency but this shift is trivial in hybrid microchannels (about ± 0.005 MHz), keeping the working frequency adjacent to the resonance frequency while supplying large enough acoustic radiation forces.

The node position can also be done by changing the thickness of the PDMS wall, attributed to the transparent behavior of PDMS against ultrasonic waves. PDMS-specific acoustic impedance is close to specific acoustic impedance of the water which implies a negligible wave reflection from the PDMS-water interface. Hence, if the distance between aluminum walls is fixed, the thickness of the PDMS wall would not affect drastically on the reflected waves, causing negligible change in the interference of the coming and reflecting waves and creating slight but controllable change in the node position. Such flexibility in the design of microchannels to obtain acoustic standing waves with desired node positions does not exist for microchannels made of acoustic-hard materials.

As shown in the acoustic simulation of the PDMS microchannel (Fig. 4e,f), acoustic standing fields in the fluid cavity are not appropriate for particle manipulation under one-displacement actuation. This means that microchannels fully made of PDMS do not offer appropriate acoustophoretic properties using one piezoelectric transducer. In practice, it is preferred to run acoustofluidic microchannels with one piezoelectric transducer located anti-symmetric relative to the fluid cavity (e.g., one-displacement actuation), knowing that working with two piezoelectric transducers need more efforts to set their electrical parameters and location relative to the fluid cavity. One of the advantages of hybrid design over the PDMS microchannel is the high acoustophoretic property when one-displacement is applied. This property was theoretically proven and experimentally tested and verified in this work.

### Acoustic energy density

To compare the acoustophoretic performance of aluminum and hybrid aluminum-PDMS microchannels, the acoustic energy density was measured from the experimental tests performed on 25 μm polystyrene particles suspended in the deionized (DI) water. Acoustic energy density (${E}_{ac}$) of an incident wave with pressure amplitude ${p}_{1}^{Amp}$ is expressed as Eq. (2)^75^.

2 $${E}_{ac}=\frac{{p}_{a}^{2}}{4{\rho }_{f}{c}_{f}^{2}}$$

Using Gor’kov expression of the acoustic radiation force, the acoustic energy density is directly proportional to the acoustic radiation force (Eq. 3)^76^

3 $$F = 4\pi a^{3} E_{ac} k\Phi \sin \left( {2kz} \right)$$

where $k=\omega /{c}_{f}$ denotes wave number, $z$ is distance between particle center and the closest velocity node, and $\Phi = f_{0} /3 + f_{1} /2$ denotes contrast factor. From Eq. (3), it is inferred that higher acoustic energy density gives larger acoustic radiation forces which determines how fast particles travel to the pressure node.

To measure acoustic energy density experimentally, the excitation frequency and the input power are set identical for all the tests. The acoustic energy density was estimated from experimental trajectories of particles^77^, considering the quasi-static motion of particles^22^. Under quasi-static motion, the acoustic radiation force $F$ is equal to Stocks drag force ${F}_{D}$, opposing the particle trajectory. In a fluid with viscosity $\eta$ and relative speed *v*, the Stocks drag force is ${F}_{D}=6\pi \eta av$, where *v* and *z* are obtained by analyzing the particle trajectory. The acoustic energy density is calculated by equalizing the acoustic radiation force with stocks’ drag force.

Theoretically, acoustic energy density is assumed to be constant across the acoustic field. However, in practice, it varies from point to point, due to manufacturing defects on the walls and assembly flaws such as uneven gluing the top cover and the transducer into the device. The acoustic energy density of five microparticles was calculated for each of the devices experimentally examined in this work. It was shown that the average energy density of the aluminum microchannel is 11 Pa at peak-to-peak voltage of 26.6 V, while this number is 2.2 Pa for the hybrid PDMS-aluminum microchannel. The difference between the acoustic energy density of the two different microchannels is attributed to the larger dissipation of vibrational energy in PDMS. These experimental values are in agreement with the numerical data reported in Tables 3 and 5, although the difference is stemmed from manufacturing or measurement errors, uncertainty in material properties, and simplification of 2D simulations limiting the planar movement of particles.

**Table 5:** The physical and mechanical properties of the materials and the liquid used for simulating aluminum, PDMS and hybrid aluminum-PDMS acoustophoretic microfluidic chips.

Parameter	Symbol			Value	Unit
**Water**					
Mass density^68^		${\rho }_{f}$	997.05		kg m^−3^
Speed of sound^68^		${c}_{f}$	1496.7		m s^−1^
Compressibility^68^		${k}_{f}$	447.7		TPa^−1^
Damping coefficient^48^		${\Gamma }_{\text{f}}$	0.004		NA
**Polystyrene**					
Mass density^78^		${\rho }_{p}$	1050		kg m^−3^
Compressibility^78^		${k}_{p}$	238		TPa^−1^
Monopole coefficient^50^		${f}_{0}$	0.468		NA
Dipole coefficient^50^		${f}_{1}$	0.034		NA
**Pyrex**					
Mass density^50^		${\rho }_{s}$	2230		kg m^−3^
Elastic modulus^50^		${C}_{11}$	69.72		GPa
Elastic modulus^50^		${C}_{44}$	26.15		GPa
Damping coefficient^50^		${\Gamma }_{\text{s}}$	0.0004		NA
**Aluminum**					
Mass density^71^		${\rho }_{s}$	2700		kg m^−3^
Elastic modulus^71^		${C}_{11}$	102		GPa
Elastic modulus^71^		${C}_{44}$	25.9		GPa
Damping coefficient^71^		${\Gamma }_{\text{s}}$	0.0013		NA
**PDMS**					
Mass density^50^		${\rho }_{s}$	1029		kg m^−3^
Elastic modulus^50^		${C}_{11}$	1.035–i0.0026		GPa
Elastic modulus^50^		${C}_{44}$	4.31–i0.68		MPa
Silicone glue’s					
Elastic modulus		$Y$	0.8		MPa
Poisson’s ratio		$\nu$	0.5		NA
Damping coefficient		${\Gamma }_{\text{s}}$	0.1		NA

Variation in particle velocity across the width of fluid cavity follows the trend of the acoustic radiation force. To compare particle velocity between the numerical model and experimental data, the velocity of four particles manipulated with 1.07 MHz were measured and compared to numerical data at the resonance frequency of 1.079 MHz in the one-displacement actuation system. Figure 7f shows velocity as a function of the width of fluid cavity in three different heights of fluid cavity (0.1 H_f_, 0.5 H_f_ and 0.9 H_f_) achieved from numerical results (green, blue and red solid curves) and four dashed curves achieved from empirically analyzing the motion of four particles obtained from experimental data. The dashed blue, orange and black curves show the velocity of three different particles above the pressure anti-node, while the dashed gray curve shows the velocity of particle under the pressure anti-node. Knowing that the nodal position in experimental tests is not located in the nodal position in the numerical model, the experimental velocity curves were first fitted based on their positions. It is seen that numerical results and experimental data have the same trend but with a small difference. Knowing that the cross section of the fluid cavity is not perfectly identical along the microchannel length, the velocity curves achieved experimentally are not well-matched for particles traveling at the same height. However, imperfections of cross section and possibly different height of particles traveling relative to the focal point of the microscope also contribute to this deviation.

Considering that the input power is related to the dissipated heat power, the level of heat generation can be estimated by measuring the temperature change in the device. We measured temperature change in the fluid cavity after 30-s switching the ultrasound signal. It was shown that the temperature change was about 1.5 °C for the aluminum microchannel while it was measured to be 8 °C for the hybrid microchannel. The higher temperature increase in the hybrid device is due to the dissipated energy in PDMS. The hybrid aluminum-PDMS microchannel works at lower temperature than the PDMS microchannel as the aluminum frame plays a role as a heat sink. The 30-s span of temperature increase in the hybrid device is still within the safe operating temperature range of the device needed for manipulation of bioparticles.

### Cost estimation of manufacturing

Most acoustophoretic devices have been made of silicon due to its desirable acoustic properties. Silicon microchannels are fabricated by lithography with a high accuracy but is expensive due to high material cost, complex and time-consuming manufacturing process, and need to cleanroom and expensive equipment. Silicon devices could be economically viable product if they are reusable. However, this is unfavorable specially for manipulation of bioparticles in different biofluids. The use of PDMS in the hybrid aluminum-PDMS design allows for complex modifications in the design that could be delivered by relatively easy and fast manufacturing techniques, high biocompatibility, and good visual clarity for visualizing bioparticles under the microscope. The hybrid aluminum-PDMS microchannel can also be reused by replacing the PDMS part (accommodating the fluid cavity) for every test, significantly reducing the cost of manufacturing and testing. The details of manufacturing cost for the hybrid aluminum-PDMS device are presented in Table S1.

## Conclusion

This work presents a numerical model and an experimental examination of a new acoustophoretic design made of a hybrid aluminum-PDMS microchannel and uses it for the manipulation of bioparticles and bead particles using acoustic standing waves. Following the implementation of proof-of-concept tests, the performance of the hybrid design was benchmarked against the aluminum microchannel. The energy density of the hybrid design was lower than the aluminum microchannel but with an appropriate magnitude of acoustic energy for successful acoustophoretic implementation. The hybrid design demonstrated to be superior to other soft or hard material designs in easily controlling the nodal position by altering the frequency. The temperature change for both devices was less than 10 °C for a 30-s acoustic activation span, which is desirable for bioparticle manipulation. The proposed hybrid design was compared to a typical silicon acoustofluidic microchannel in terms of manufacturing complexity and cost. The cost of hybrid design was significantly lower than the standard lithography methods while also provided the opportunity of reusability with simple manufacturing steps. Considering the benefits of the hybrid design, it is potentially desirable for developing the next generation of economically viable acoustophoretic products for ultrasound particle manipulation in bioengineering applications.

## Materials and methods

### Design and material of the microchannel

Acoustophoretic devices generally have a central microchannel consists of a fluid cavity with a rectangular cross section, width W_f_ and height H_f_ (Fig. 1a–c). The fluid cavity Ω_f_ is surrounded by an elastic solid Ω_E_. The solid domain has two parts: first substrate Ω_s_ in which the fluid cavity mounts and second ceiling Ω_Py_. An ultrasonic transducer is used to generate acoustic waves. This ultrasonic transducer could be, for example, a lead zirconate titanate (PZT) piezoelectric attached beneath the substrate in an anti-symmetric position relative to the centerline of fluid cavity (y axis). Alternatively, a two piezoelectric anti-phase actuation configuration can be used. A periodic displacement is modeled in the simulation to represent the transducer. In this work, both one and two piezoelectric actuations are numerically modeled by applying one-displacement actuation (Fig. 1b) and two-displacement actuation (Fig. 1c), respectively^50^.

This work focuses on microchannels made of aluminum, polydimethylsiloxane (PDMS), and hybrid PDMS-aluminum channel walls while the channel ceiling is made of a flat borosilicate glass lid (Pyrex). In aluminum microchannels, a silicone adhesive was used to adhere the Pyrex lid to the aluminum substrate, hence the glue layer was defined as one extra domain in the acoustic simulation, modeled as a thin elastic layer. The microchannel entirely made of aluminum was used as a basis to compare acoustic performance of devices made of other materials. Following the computational analysis of all microchannels, the aluminum microchannel and hybrid aluminum-PDMS microchannel microfluidic chips were manufactured and experimentally tested for acoustic manipulation of particles.

### Theory of acoustophoretic manipulation of particles

For acoustophoretic microfluidic devices, acoustic waves are generated by time-harmonic displacements produced by a piezoelectric transducer excited by AC electric signals. The wavy motion is convoyed to the substrate and the wave passes through the walls, enters the fluid cavity and reflects between the cavity walls, making a standing acoustic pressure field in the fluid. In the presence of a particle in the fluid, the first-order pressure field is scattered by its surface, disturbing the pressure field in vicinity of the particle and generating the primary acoustic radiation force. This force pushes the particle toward a pressure wave node or anti-node depending on contrast factor of the particle. In the presence of more than one particle, the scattering pressure wave stemmed by other particles imposes inter-particle forces or secondary radiation forces. In low suspension of particles, where the distance among particles is much greater than particle radius $a$, the secondary radiation force is considered negligible^25^.

To model dynamic behavior of the acoustophoretic microfluidic system, the fields whether velocity or pressure are modeled as $A\left(r\cdot t\right)=A\left(r\right){e}^{-i\omega t}$, where $A\left(r,t\right)$ is determined field and $\omega$ and t are angular frequency and time, respectively. $A\left(r\right)$ is space-dependent amplitude and ${e}^{-i\omega t}$ is a phase factor considering time-harmonic behavior of the system. Since time-harmonic displacements are very small, the device behavior is modeled by linear acoustic equations, of which the phase factor is eliminated. In solids with density ${\rho }_{s}$, the mechanical equilibrium under harmonic loading is defined as Eq. (4)

4 $$\nabla \cdot {\varvec{\upsigma}}=-{\rho }_{s}{\omega }^{2}\mathbf{u}$$

where $\mathbf{u}$ and ${\varvec{\upsigma}}$ are displacement and stress tensor, respectively. The damping effects are considered by multiplying $\left(1+\text{i}{\Gamma }_{\text{s}}\right)$ to Eq. (4), where ${\Gamma }_{\text{s}}$ is damping of the solid domain^48^.

Knowing that in the aluminum microchannel, the Pyrex lid is attached to the substrate by a layer of silicone glue, the glue layer is considered in the modeling. Because of the high cost of simulating this thin layer, it was approximated by a thin elastic layer constituting spring constants of the glue^48^. Considering $\nu$ and Y as poisson’s ratio and Young’s modulus, respectively, the spring constants in normal ${k}_{gl}^{n}$ and tangent ${k}_{gl}^{t}$ directions for a glue layer with thickness ${l}_{gl}$ are defined in Eqs. (5) and (6):

5 $${k}_{gl}^{n}=\frac{1}{{l}_{gl}}\frac{Y \left(1-\nu \right)}{\left(1+\nu \right)\left(1-2\nu \right)}$$

6 $${k}_{gl}^{t}=\frac{1}{{l}_{gl}}\frac{Y }{2\left(1+\nu \right)}$$

Other solid domains of the microfluidic device are modeled as a linear elastic and isotropic material. The relation between stress and displacement is defined as Eq. (7)^79^

7 $$\left(\begin{array}{c}{\sigma }_{xx}\\ {\sigma }_{yy}\\ {\sigma }_{zz}\\ {\sigma }_{yz}\\ {\sigma }_{xz}\\ {\sigma }_{xy}\end{array}\right)=\left[\begin{array}{c}{C}_{11}\\ {C}_{12}\\ {C}_{12}\\ 0\\ 0\\ 0\end{array} \begin{array}{c}{C}_{12}\\ {C}_{11}\\ {C}_{12}\\ 0\\ 0\\ 0\end{array} \begin{array}{c}{C}_{12}\\ {C}_{12}\\ {C}_{11}\\ 0\\ 0\\ 0\end{array} \begin{array}{c}0\\ 0\\ 0\\ {C}_{44}\\ 0\\ 0\end{array} \begin{array}{c}0\\ 0\\ 0\\ 0\\ {C}_{44}\\ 0\end{array} \begin{array}{c}0\\ 0\\ 0\\ 0\\ 0\\ {C}_{44}\end{array}\right]\left(\begin{array}{c}{\partial }_{x}{u}_{x}\\ {\partial }_{y}{u}_{y}\\ {\partial }_{z}{u}_{z}\\ {\partial }_{y}{u}_{z}+{\partial }_{z}{u}_{y}\\ {\partial }_{x}{u}_{z}+{\partial }_{z}{u}_{x}\\ {\partial }_{x}{u}_{y}+{\partial }_{y}{u}_{x}\end{array}\right)$$

wherein for an isotropic material in this stiffness tensor, ${C}_{11}$ and ${C}_{44}$ are independent and ${C}_{12}={C}_{11}-2{C}_{22}$. The harmonic displacement of fluid cavity affects pressure distribution. For an inviscid fluid with density ${\rho }_{f}$, speed of sound ${c}_{f}$ and compressibility ${k}_{f}={\left({\rho }_{f}{c}_{f}^{2}\right)}^{-1}$, the first-order pressure field ${p}_{1}$ and velocity field ${\text{v}}_{1}$ are modeled by Helmholtz equations.

8 $${\nabla }^{2}{p}_{1}=-{k}_{f}{p}_{1}$$

9 $${\text{v}}_{1}=\frac{i}{\omega {\rho }_{f}}\nabla {p}_{1}$$

The damping of the fluid domain is considered by multiplying $(1+i{\Gamma }_{f})$ to Eq. (9)^48^, where ${\Gamma }_{f}$ is damping coefficient of the fluid. The first-order pressure field produces the primary acoustic radiation force on particles. The primary acoustic radiation force ($F$) applied on particles with a small viscous boundary layer compared to their radius $a$, with compressibility ${k}_{p}$ and density ${\rho }_{p}$, is estimated by monopole ${f}_{0}$ and dipole ${f}_{1}$ scattering coefficients (Eqs. 10 and 11)^80^.

10 $$F=-\pi {a}^{3}\left[\frac{2}{3}{k}_{f}Re\left({f}_{0}^{*}{p}_{1}^{*}\nabla p\right)-{\rho }_{f}Re\left({f}_{1}^{*}{\text{v}}_{1}^{*}\cdot \nabla {\text{v}}_{1}\right)\right]$$

11 $${f}_{0}=1-\frac{{k}_{p}}{{k}_{f}}\;{\text{and}}\;{f}_{1}=\frac{2\left({\rho }_{p}-{\rho }_{f}\right)}{2{\rho }_{p}+{\rho }_{f}}$$

The asterisk sign is a complex conjugate. The spatial average of the acoustic radiation forces is given by Eqs. (12) and (13)^50^.

12 $$\overline{F}_{z} = \frac{1}{{W_{f} H_{f} }}\int\limits_{{\Omega_{f} }} {\frac{z}{\left| z \right|}F_{z} \;dydz}$$

13 $$\overline{F}_{y} = \frac{1}{{W_{f} H_{f} }}\int\limits_{{\Omega_{f} }} {F_{y} \;dydz}$$

The strength of acoustic pressure field depends on the device performance, first in converting electrical energy into mechanical energy in form of time-harmonic displacement, and second in conveying mechanical energy to the fluid, affecting the first order pressure. Thus, density of the acoustic energy in the solid (${E}_{s}$) and the fluid (${E}_{f}$) (Eqs. 15 and 16, respectively) is a criterion to evaluate the device performance (Eq. 13)

14 $${E}_{s}=\frac{1}{2}{\rho }_{s}{\omega }^{2}\langle {u}_{j}{u}_{j}\rangle +\frac{1}{2}\langle {\gamma }_{ij}{\sigma }_{ij}\rangle$$

15 $${E}_{f}=\frac{1}{2}{\rho }_{f}\langle {\text{v}}_{j}{\text{v}}_{j}\rangle +\frac{1}{2}{k}_{f}\langle {p}_{1}^{2}\rangle$$

where $\langle AB\rangle =\frac{1}{4}\left({A}^{*}B+A{B}^{*}\right)$ is time-average of A and B fields over an oscillation and ${\gamma }_{ij}=\frac{1}{2}\left({\partial }_{i}{u}_{j}+{\partial }_{j}{u}_{i}\right)$ determines components of the strain tensor. Therefore, the acoustic energy stored in the solid and the fluid are obtained by Eq. (16).

16 $$\varepsilon_{s} = \int\limits_{{\Omega_{s} }} {E_{s} \;dydz} ,\quad \varepsilon_{f} = \int\limits_{{\Omega_{f} }} {E_{f} \;dydz}$$

The boundary conditions between the solid and the fluid domains are continuity of velocity and stress field. Knowing that the boundaries between the solid and air are stress free, the normal stress on these boundaries is set to zero. To simulate the piezoelectric behavior, the normal displacement is applied on substrate’s boundaries, where Eqs. (17a–17c) represent boundary conditions applied on the interface with normal vector $\mathbf{n}$:

17a $${\text{Solid and fluid interface }}\left\{\begin{array}{l}\mathbf{v}\cdot \mathbf{n}=-i\omega \mathbf{u}\cdot \mathbf{n}\\ {{\varvec{\upsigma}}}_{sl}.\mathbf{n}=-pn\end{array}\right.$$

17b $${\text{Solid and air interface }}{{\varvec{\upsigma}}}_{sl}\cdot \mathbf{n}=0$$

17c $${\text{Displacement actuation }}\mathbf{u}={d}_{0}\mathbf{n}$$

### Numerical analysis

Considering the governing harmonic loadings in both the solid and liquid (Eqs. 4 and 8) and with boundary conditions of Eqs. (17a–17c), the dynamic behavior of acoustic device was modeled in two-dimensional (2D) and in a Cartesian coordinate system (with the origin at (Wf/2, Hf/2)) by COMSOL Multiphysics software. Different materials of microchannels were modeled in this study, including a microchannel fully made of aluminum, a microchannel fully made of PDMS, and a microchannel made of hybrid aluminum-PDMS. To reduce the computation cost, behavior of the piezoelectric material is ignored in the simulation. Also, mesh convergence analysis is performed using the procedure explained in Ref.^81^.

In resonance frequencies, maximum pressure amplitude ${p}_{1}^{Amp}$ and consequently maximum acoustic radiation force ${F}_{z}^{Amp}$ are generated, which are the ideal conditions for manipulating microparticles with high energy efficiency. For a microchannel with the width of fluid cavity ${W}_{f}=700$ μm, the resonance frequency with anti-symmetric pressure field (${W}_{f}={\lambda }_{f}/2$) in an infinitely hard-wall fluid cavity filled with the water is obtained as ${f}_{hard}=\frac{{c}_{f}}{2{W}_{f}}\approx 1.07$ MHz, where ${f}_{hard}$ is resonance frequency of the device and ${c}_{f}$ is wave’s speed in the medium. In a fluid domain with infinitely hard-walls, actuation with ${f}_{hard}$ gives an ideal half sinusoidal pressure wave distribution along the width of fluid cavity with a maximum pressure applied on the walls and a nodal plane in the centerline of channel’s width. However, the surrounding domains around the fluid cavity in the experiments affect pressure distribution in the fluid cavity. The more the acoustically harder materials used for manufacturing of acoustic devices, the more similar the pressure distribution in the fluid cavity to the ideal condition. The higher the specific acoustic impedance of the hard walls relative to the water, the higher the intensity reflection coefficient. Hence, most of the acoustic wave energy reflects from the walls and interferes with the incident acoustic wave, generating a standing wave with a large pressure amplitude.

Aluminum is approximately a sound-hard material with specific acoustic impedance of $17.33\times {10}^{6}$ kg m^−2^ s^−1^, close to silicon’s specific acoustic impedance of $19.79\times {10}^{6}$ kg m^−2^ s^−1^ with a widespread usage in manufacturing of BAW microfluidic chips. However, aluminum is less expensive and a good choice to replace silicon. In this work, aluminum was used as a sound-hard material. In aluminum microchannels studied in this work, the walls of fluid cavity are made of aluminum while the ceiling is made of a Pyrex lid (Fig. 1a). The Pyrex lid was attached to the substrate using a 50 µm thick silicone glue. The damping property of the glue layer at vibrations in MHz range was not reported neither in the literature nor by the manufacturers, wherein we consider damping factor 0.1 for this material^75^. The glue layer was modeled by a thin elastic layer resembling an elastic coupling. The aluminum substrate and the Pyrex lid were modeled as elastic solid domains. The material properties and dimensions of all parts are given in Tables 5 and 6. For ease of manufacturing and setting up the experimental system as well as controlling the temperature, the dimensions of the solid domains were set to be much larger than 1/4 λ. The piezoelectric actuation was modeled by a normal displacement actuation with the amplitude of 0.1 nm applied on the substrate’s wall.

**Table 6:** Geometrical parameters of different microchannels used in this acoustophoretic study.

Parameter	Symbol	Value (mm)
Height	H_Py_	1
Height	H_s_	2
Actuator gap	ΔW	0.1
Width	W_*f*_	0.7
Height	H_*f*_	0.3
**Aluminum microchannel**		
Width	W_Py_	9.7
**PDMS microchannel**		
Width	W_Py_	3.06
**Hybrid aluminum-PDMS microchannel**		
Width	W_Py_	23.58
Width	W_PDMS_	2.58

### Cell culture

Human breast cancer cell lines used in acoustophoretic experiments were BT-20 and MDA-MB-231 breast cancer cells, provided by Pasteur Institute (Tehran, Iran). The cells were cultured in DMEM supplemented with 10% fetal bovine serum (FBS) and 1% penicillin–streptomycin antibiotics at 37 °C in a humidified incubator containing 5% CO_2_. The cells were washed with phosphate buffer saline (PBS) after reaching the confluency of 75–80%, then harvested with 0.025% trypsin–0.01% ethylenediaminetetraacetic acid (EDTA). The trypsin was then deactivated and the cells after being centrifuged at 1500 rpm for 5 min were suspended in a new culture medium, and either passaged or applied for the experiments.

### Acoustophoretic testing

Following the design and simulation of acoustophoretic models for aluminum, PDMS, and hybrid microchannels, these microfluidic systems were fabricated and tested experimentally and compared to numerical data. Aluminum and PDMS were the materials of choice for fabrication of ultrasound-powered microfluidic devices in this work, knowing their low manufacturing cost and high accessibility compared to silicon microchips. The aluminum microchannels were produced by a 3-axis CNC milling machine (model: KNS 2015). For a proof-of-concept test, the fluid cavity in the acoustic field was selected to be a straight slot similar to the designs reported in the literature^82,83^. The fluid inlet and outlet were placed at the two ends of fluid cavity. The top cover of all devices was set to Pyrex lid. The substrate is an aluminum sheet glued to the microchannel by a silicone glue. Particle-tracing observation was implemented by an optical microscope (Leica DM IL LED). A piezoelectric transducer (Pz26 from Meggitt’s Ferroperm Piezoceramic) enabling one-displacement actuation was located on one side of the fluid cavity and used to consistently generate sound for all the devices while it was epoxy glued to the setup.

For fabrication of the hybrid aluminum-PDMS microchannel, the PDMS domain was first produced from the straight slot aluminum mold. An aluminum mold along with an aluminum frame was manufactured by 3-axis CNC milling machine. The aluminum frame was assembled on the aluminum mold to form the outer boundary of the PDMS domain. The PDMS and its curing agent was mixed with the ratio 10:1, poured onto the mold/frame assembly and cured for 30 min in 80 °C. The cured PDMS layer and the frame were peeled off, surface-treated by an oxygen plasma, and bonded to the lid for a seamless assembly. The piezoelectric transducer was connected to a signal function generator (MEGATEK model MFG-2120) and a custom-made amplifier. The experiments were conducted for manipulation of BT-20 and MDA-MB-231 breast cancer cells suspended in PBS (5% w/w) as well as polystyrene beads (diameter: 25 μm, 5% w/w) suspended in deionized (DI) water. To prevent particle aggregation or attachment to the channel walls, 4% Pluronic F-127 surfactant was added to the suspension. In all the experiments, the fluid was stationary prior to switching on the ultrasound actuation to eliminate the effect of drag force on particle movement, allowing for accurate particle traveling to the pressure nodes.

## Supplementary Information

Supplementary Information.

Supplementary Video 1.
