# Supplementary material for: Numerical and experimental analysis of a hybrid material acoustophoretic device for manipulation of microparticles
Source: Sci Rep. 2021 Nov 11;11:22048. doi: 10.1038/s41598-021-01459-0 (PMC8586004; doi:10.1038/s41598-021-01459-0)
Supplement: Supplementary file 1 — Supplementary Information. [file 41598_2021_1459_MOESM1_ESM.pdf]

Numerical and experimental analysis of a hybrid material acoustophoretic device for manipulation of microparticles

Alireza Barani,[a,b] Peiman Mosaddegh,[a] Shaghayegh Haghjooy Javanmard,[b] Shahrokh Sepehrirahnama[c] and Amir Sanati-Nezhad[d]

[a] Department of Mechanical Engineering, Isfahan University of Technology, Isfahan, 84156-83111, Iran.
[b] Applied Physiology Research Center, Cardiovascular Research Institute, Isfahan University of Medical Science, Isfahan, 81746-73461, Iran.
[c] Mechanical Engineering Department, National University of Singapore, 9 Engineering Drive 1, Singapore 117575.
[d] BioMEMS and Bioinspired Microfluidic Laboratory, Department of Mechanical and Manufacturing Engineering, University of Calgary, MEB 214 2500 University Drive NW, Calgary, Alberta T2N1 N4 Canada

Supplementary Information

The cost of the hybrid aluminum-polydimethylsiloxane (PDMS) device was compared to the cost of making one silicon microchannel for acoustophoretic applications[1] though other works reported more complex processes and expensive costs for the silicon chips.[2,3] Although personnel costs are not included in calculations (although the personal costs might be a dominant cost share in some settings), these costs are obviously larger for manufacturing of silicon microchannels given the need for sophisticated machines and high-tech expertise.

Table S1. The cost and manufacturing steps of making hybrid aluminum-PDMS and silicon microchannels (excluding personnel cost).

**Hybrid aluminum-PDMS chip**	**Cost (USD)**	**Silicon chip [1] (glass-Si-glass)**	**Cost (USD)**
**Microchannel Parts**			
Pyrex lid (76 × 26 × 1 mm)	0.032	Glass wafer X2 (50.8×50.8×0.5 mm)	12.5
Aluminum frame +PDMS mold	0.03	Silicon wafer (50.8×50.8×0.3 mm)	16.9
PDMS	0.095	Mask	130
-	-	Connectors (made of PDMS)	0.08
**Total**	0.157	Total	159.48
**Microchannel Manufacturing Steps**			
- Aluminum frame (CNC milling)<br>- PDMS mold (CNC milling)<br>- Aluminum frame and mold (cleaning)<br>- PDMS (molding and curing)<br>- Glass and PDMS bonding (plasma)<br>- Punching (inlets and outlets)<br>- Aluminum frame assembly		- Wafer cleaning (RCA)<br>- Photoresist (spin and bake)<br>- Mask (align and expose)<br>- Photoresist (develop)<br>- Etch<br>- Photoresist (remove)<br>- Drilling inlets and outlets<br>- Wafer bonding (anodic)<br>- Dicing<br>- Connectors	

C

1 Samarasekera, C. *Variations on Acoustophoretic Microchannels: From the Facile to the Highly Capable* PhD thesis, University of Waterloo, (2018).
2 Leibacher, I., Schatzer, S. & Dual, J. Impedance matched channel walls in acoustofluidic systems. *Lab Chip,* **14**, 463-470, 2017.
3 Qi, Z. B. *et al.* Disposable silicon-glass microfluidic devices: precise, robust and cheap. *Lab Chip* **18**, 3872–3880 (2018).
